# Midkine Promotes Tumor Growth and Attenuates the Effect of Cisplatin in Small Cell Lung Cancer

**DOI:** 10.1002/cam4.71034

**Published:** 2025-07-07

**Authors:** Shotaro Ito, Jun Sakakibara‐Konishi, Mineyoshi Sato, Tetsuaki Shoji, Megumi Furuta, Hirofumi Takahashi, Kosuke Tsuji, Daisuke Morinaga, Masahiro Kashima, Hidenori Kitai, Junko Kikuchi, Eiki Kikuchi, Kanako C. Hatanaka, Yutaka Hatanaka, Kyoko Hida, Takuro Noguchi, Satoshi Konno

**Affiliations:** ^1^ Department of Respiratory Medicine Faculty of Medicine, Hokkaido University Sapporo Japan; ^2^ Center for Development of Advanced Diagnostics Hokkaido University Hospital Sapporo Japan; ^3^ Department of Vascular Biology and Molecular Pathology Hokkaido University Faculty of Dental Medicine Sapporo Japan; ^4^ Department of Medical Oncology Faculty of Medicine and Graduate School of Medicine, Hokkaido University Sapporo Japan

**Keywords:** AKT, biomarker, Midkine, small cell lung cancer, therapeutic target, tumor progression

## Abstract

**Purpose:**

Small cell lung cancer (SCLC) is a highly aggressive disease associated with poor patient survival rates. The addition of an anti‐programmed death ligand 1 antibody to platinum combination chemotherapy can improve its prognosis. However, only a few patients achieve a long‐term response; thus, establishing new therapies for SCLC is crucial. Midkine (MDK) is a heparin‐binding growth factor involved in various biological processes, including cell proliferation and chemotherapeutic resistance, in diverse cancers. MDK has garnered attention as a therapeutic and diagnostic target for several cancers; however, only a few studies have evaluated its expression and function in SCLC. This study aimed to evaluate the MDK expression in human SCLC tissue and human SCLC cell lines, and to clarify its function in tumorigenesis.

**Methods:**

MDK expression was analyzed in vitro and in vivo through ELISA, immunohistochemistry, and western blotting. Its effects on cell proliferation, as well as the effects of cisplatin, were evaluated using the MTT assay.

**Results:**

MDK was pathologically expressed in human SCLC tumor tissues but not in normal lung tissues. Serum MDK concentrations in patients with SCLC reflected the SCLC tumor burden and were correlated with response to treatment. Moreover, MDK induced cell proliferation and attenuated the effects of cisplatin in SCLC cell lines. An MDK inhibitor and cisplatin exerted synergistic antitumor effects both in vitro and in vivo. Furthermore, MDK positively regulated the AKT pathway.

**Conclusion:**

Our findings indicate that MDK promotes cell proliferation and chemotherapeutic resistance by activating the AKT pathway in SCLC cells. Therefore, MDK may be a potential therapeutic and diagnostic target for SCLC.

## 1 Introduction

Small cell lung cancer (SCLC), which accounts for approximately 14% of all lung cancer cases, is an aggressive disease that is most often diagnosed at its metastatic stages, correlating with the poor survival rates in patients affected by it [1]. Platinum‐based chemotherapy is the standard treatment for extensive‐stage SCLC, eliciting favorable responses in most cases; however, patients have been reported to rapidly develop therapeutic resistance to platinum‐based regimens [2]. In addition, it has been reported that adding an anti‐programmed death ligand 1 antibody to platinum combination chemotherapies can improve the median survival rate [3, 4]. However, three‐year survival and five‐year overall survival estimates are approximately 17% and 12%, respectively, in two phase III clinical trials on the combined use of platinum chemotherapy and immune checkpoint inhibitors for SCLC treatment, and effective long‐term response has only been reported in a few patients [5, 6].

SCLC subtypes have recently been characterized and defined based on the relative expression of the genes, *ASCL1*, *NEUROD1*, *POU2F3*, and *YAP1*, which encode four major transcriptional regulators. While SCLC with upregulated achaete‐scute family bHLH transcription factor 1 (ASCL1) or neuronal differentiation 1 (NEUROD1) expression is a classic type with a neuroendocrine (NE) feature, other SCLC subtypes lack NE features, making SCLC a highly heterogeneous tumor [7, 8, 9, 10, 11]. The neurogenic locus notch homolog protein (Notch) pathway and Myc play important roles in SCLC heterogeneity [12, 13]. Although each subtype exhibits specific drug vulnerabilities [14, 15, 16, 17], targeted therapies for SCLC are still not well established. Tarlatamab, a bispecific T‐cell engager immunotherapeutic agent targeting delta‐like ligand 3 (DLL3) and CD3, was recently approved for the treatment of previously treated SCLC patients. However, data clearly showing the association between DLL3 expression and different treatment outcomes is unavailable, and further validation is needed to determine which patients could be expected to benefit from this therapy [18].

Midkine (MDK), a heparin‐binding growth factor, was first identified as a highly expressed protein during mouse embryogenesis [19]. MDK is a multifunctional secreted protein that interacts with various plasma membrane receptors [20]. It is highly expressed in various malignancies and participates in cell proliferation, apoptosis evasion, and tumor microenvironment (TME) rewiring for immune resistance by activating intracellular pathways [20, 21, 22, 23, 24, 25, 26]. Therefore, MDK has gained attention as a therapeutic target [27, 28]. In SCLC, MDK, produced by non‐NE SCLC cells, promotes NE SCLC cell survival [12]. Moreover, MDK is associated with resistance to cisplatin (CDDP) and immune checkpoint inhibitors (ICIs) and is a potential therapeutic target for SCLC [25, 26, 29, 30]. However, only a few studies have evaluated its expression and function in SCLC. Thus, in this study, we investigated MDK expression and function in SCLC.

## 2 Materials and Methods

### 2.1 Reagents

The MDK inhibitor (iMDK) was purchased from Merck (508,052, Merck, Darmstadt, Germany), dissolved in DMSO, and stored at −30°C until further use. Recombinant human MDK (rhMDK) (450‐16, Peprotech, RockyHill, NJ, USA) was purchased, dissolved in nuclear‐free water at a concentration of 100 μg/mL, and stored at −80°C until further use. The AKT inhibitor (iAKT) was purchased from Selleck Biotech (MK2206, Selleck Biotech, Kanagawa, Japan), dissolved in DMSO, and stored at −80°C until further use.

### 2.2 Cell Lines and Establishment of CDDP‐Resistant Lung Cancer Cell Lines

We used nine SCLC cell lines in this study. SBC5, SBC3, MS1L, and RERF‐LC‐MA were procured from the Japanese Collection of Research Bioresources Cell Bank (Osaka, Japan), while H592, H1688, H209, H82, and H69 were purchased from the American Type Culture Collection (Manassas, VA, USA). SBC5, SBC3, and RERF‐LC‐MA were maintained in minimum essential medium, while MS1L, H1688, H592, H209, H82, and H69 were maintained in RPMI 1640 medium supplemented with 10% fetal bovine serum and 100 U/mL penicillin‐streptomycin in a humidified atmosphere at 37°C with 5% CO_2_. Because of the quality control issues associated with RERF‐LC‐MA cells, only subgroup markers, MDK expression, and Notch signaling were evaluated in these cells in conjunction with other cell lines. Based on data from a previous study, the CDDP‐resistant SCLC cell lines, SBC5R and SBC3R, were established from the parent cell lines, SBC5 and SBC3, respectively. The parent cell lines were treated with gradually increasing CDDP concentrations (maximum 2 μmol/L), and then cultured in a medium containing 2 μmol/L CDDP for 3 months.

### 2.3 ELISA for Human MDK and Tumor Burden Evaluation

Blood samples were collected from patients with SCLC at the Hokkaido University Hospital before first‐line chemotherapy, after two and four cycles of chemotherapy, and during tumor progression, between April 2020 and December 2023. Histopathological diagnoses were made by pathologists at the Pathological Institute of Hokkaido University Hospital. In addition, blood samples were collected from 10 healthy controls (median age: 35.6) with no history of malignancy. All individuals provided signed informed consent regarding the publishing of their data. Furthermore, conditioned medium from the SCLC cell lines was collected and stored with these blood samples at −30°C until evaluation. Human serum MDK concentrations (s‐MDK) and MDK concentrations in the culture medium were measured using an ELISA kit for human MDK (ab193761; Abcam, Sydney, Australia) according to the instructions of the manufacturer. Optical density was measured at 450 nm using a microplate reader (Varioskan Flash; Thermo Fisher Scientific, Waltham, MA, USA); using this data, a receiver operating characteristic (ROC) curve was established, and the corresponding area under the curve (AUC) values were calculated. Using the ROC curve, an optimized cutoff value (53.7 pg/mL) was defined for s‐MDK with respect to sensitivity and specificity. In patients with SCLC, high‐ and low‐MDK expression groups were defined using this s‐MDK cutoff value. Tumor volume was calculated as the baseline sum of the longest diameter (BSLD) of the target lesion [31]. BSLD was calculated by measuring the target lesions based on the Response Evaluation Criteria for Solid Tumors Version 1.1, and the median (BSLD: 80.12 mm) was used as the cutoff value to classify patients into the high‐ and low‐BSLD groups.

### 2.4 Immunohistochemistry (IHC)

ASCL1, NEUROD1, POU class 2 homeobox 3, yes‐associated protein 1, synaptophysin (SYP), RE1 silencing transcription factor (REST), and MDK expression in human tumor tissues from four patients with SCLC was analyzed through IHC. In addition, MDK and P‐AKT expression in dissected xenograft tumors was analyzed through IHC. The IHC scores for MDK and P‐AKT were evaluated and calculated based on the staining intensity and proportion. The staining intensity was stratified as zero (negative), one (weak), two (moderate), or three (strong). The proportion of the positive staining area was scored from 0% to 100%. Finally, the IHC score, ranging from 0 to 300, was calculated by multiplying the values for the staining intensity and staining proportion. Next, 5‐μm thick tumor tissue sections made from paraffin‐embedded blocks were subjected to IHC staining using the specific antibodies listed in Table S1.

### 2.5 Antibodies and Western Blotting

Whole‐cell lysates were subjected to western blotting and analyzed for protein levels using the specific antibodies listed in Table S2. Band intensity was calculated through quantitative densitometric analyses using the ImageJ ver. 1.53 software (National Institutes of Health, Bethesda, MD, USA). Standardization was performed by measuring actin levels in the same blots using anti‐actin antibodies (1:1500; A2066, Sigma‐Aldrich, St. Louis, MO, USA). The experiments were performed in triplicate.

### 2.6 qRT‐PCR

Total RNA was isolated using the Fast Gene RNA Premium Kit (Nippon Genetics) according to the instructions of the manufacturer. RNA was reverse‐transcribed into cDNA using TaqMan reverse transcription reagents and random hexamers (Applied Biosystems). mRNA expression was determined via qRT‐PCR using the StepOnePlus Real‐Time PCR System (Applied Biosystems) according to the instructions of the manufacturer. The TaqMan Universal PCR Master Mix, with MDK and glyceraldehyde‐3‐phosphate dehydrogenase (*GAPDH*) assays (Applied Biosystems), was used. The mean expression of each gene was determined relative to that of *GAPDH*. MDK mRNA expression was assessed using TaqMan's primer Hs00171064_m1. The primer probe sequences for the TaqMan assay are private and cannot be shared. Nonetheless, the product website (https://www.thermofisher.com/taqman‐gene‐expression/product/Hs00171064_m1?CID=&ICID=&subtype=) gives a rough idea of the section of the MDK sequence that this primer recognizes. All experiments were performed in triplicate. Data are presented as the mean ± standard deviation. Statistical significance was set at *p* < 0.05.

### 2.7 MDK Overexpression

The human complementary DNA (cDNA)‐open reading frame (ORF) clone of MDK (RG221818, ORIGENE), the blank vector (PS100019, ORIGENE), and TurboFectin 8.0 (transfection reagent) were purchased from OriGene Technologies (Rockville, MD, USA). Plasmid construction was evaluated using following primers: VP1.5 Forward: 5′ GGACTTTCCAAAATGTCG 3′ and XL39 Reverse: 5′ ATTAGGACAAGGCTGGTGGG 3′. Using gel electrophoresis, we investigated whether plasmids generated from RG221818 and PS100019 retained their target genes. An image of the gel is shown in Figure 3B. SBC3 and MS1L cells were divided into two equal groups that is, the MDK‐overexpressing (transfected with the MDK‐ORF plasmid) and control (transfected with pCMV6‐entry) groups. The day before transfection, SBC3 (3 × 10^5^/well) and MS1L (5 × 10^5^/well) cells were seeded onto six‐well plates, and then transfected with 2 μg of the MDK‐ORF plasmid or vector in serum‐free Opti‐minimal essential medium (MEM) I (Thermo Fisher Scientific) and 12 μL of TurboFectin 8.0. After 24 h, the transfected cells were diluted at a 1:10 ratio in 10‐cm dishes, and the culture medium was replaced with complete medium containing G418 (600 μg/mL). The culture was screened using G418 (Thermo Fisher Scientific) to obtain stable and positive clones.

### 2.8 Cell Proliferation Assay

Cell growth was assessed using the MTT cell proliferation assay according to the instructions of the manufacturer (Thermo Fisher Scientific), and absorption at 560 nm was determined using a microplate reader. SCLC cells were seeded onto 96‐well plates containing rhMDK at a density of 1 × 10^3^/well. Then, rhMDK was administered at the time of cell seeding and on the day after seeding. ShMDK‐overexpressing cells were seeded onto 96‐well plates at a density of 1 × 10^3^/well for 96 h and then subjected to the MTT assay. The cytotoxicity of each drug was assessed. Tumor cells were seeded onto 96‐well plates at a density of 5–20 × 10^3^ (SBC5 and SBC3: 5 × 10^3^, MS1L and H82: 10 × 10^3^, and H69: 20 × 10^3^)/well for 24 h. The medium was replaced with fresh medium containing each drug. Then, the cells were treated for 72 h, and IC_50_ was calculated based on the results (Tables S3 and S4). Synergism between CDDP and iMDK was evaluated by performing the combination index analysis adapted from the median–principle methods described by Chou and Talalay [32] using CalcuSyn 2.1 (Biosoft, Bengaluru, India). The experiments were performed in triplicate.

### 2.9 Xenograft Mouse Model

SBC5 control and SBC5 small hairpin (sh) RNA cells (5 × 10^6^ cells) were diluted in 200 μL of PBS and subcutaneously injected into the right posterior legs of athymic 5‐week‐old female nude (nu^+^/nu^+^) mice. Tumor dimensions were measured daily using digital calipers, and the tumor volume was calculated as:

Tumor volume = width^2^ × length/2 [33].

Drug treatments were initiated when the mean tumor volume reached approximately 150–250 mm^3^. Body weights and general conditions were monitored. CDDP was administered by intraperitoneal injection once a week (1 mg/kg/day), and iMDK was administered by intraperitoneal injection thrice a week (9 mg/kg/day) [28]. Each group consisted of four mice, and two independent experiments were performed.

### 2.10 Statistical Analyses

All data were derived from at least three independent experiments and are presented as the mean ± standard deviation, unless otherwise indicated. Differences between groups were analyzed using Welch's t‐test and multiple comparison tests. Statistical significance was set at *p* < 0.05. Statistical analyses were performed using GraphPad Prism v.10.0 (GraphPad Software, San Diego, CA, USA). Additional methods are detailed in Document S1.

## 3 Results

### 3.1 s‐MDK Levels Are Elevated in Patients With SCLC and Increase With Cancer Progression

**FIGURE 1:**
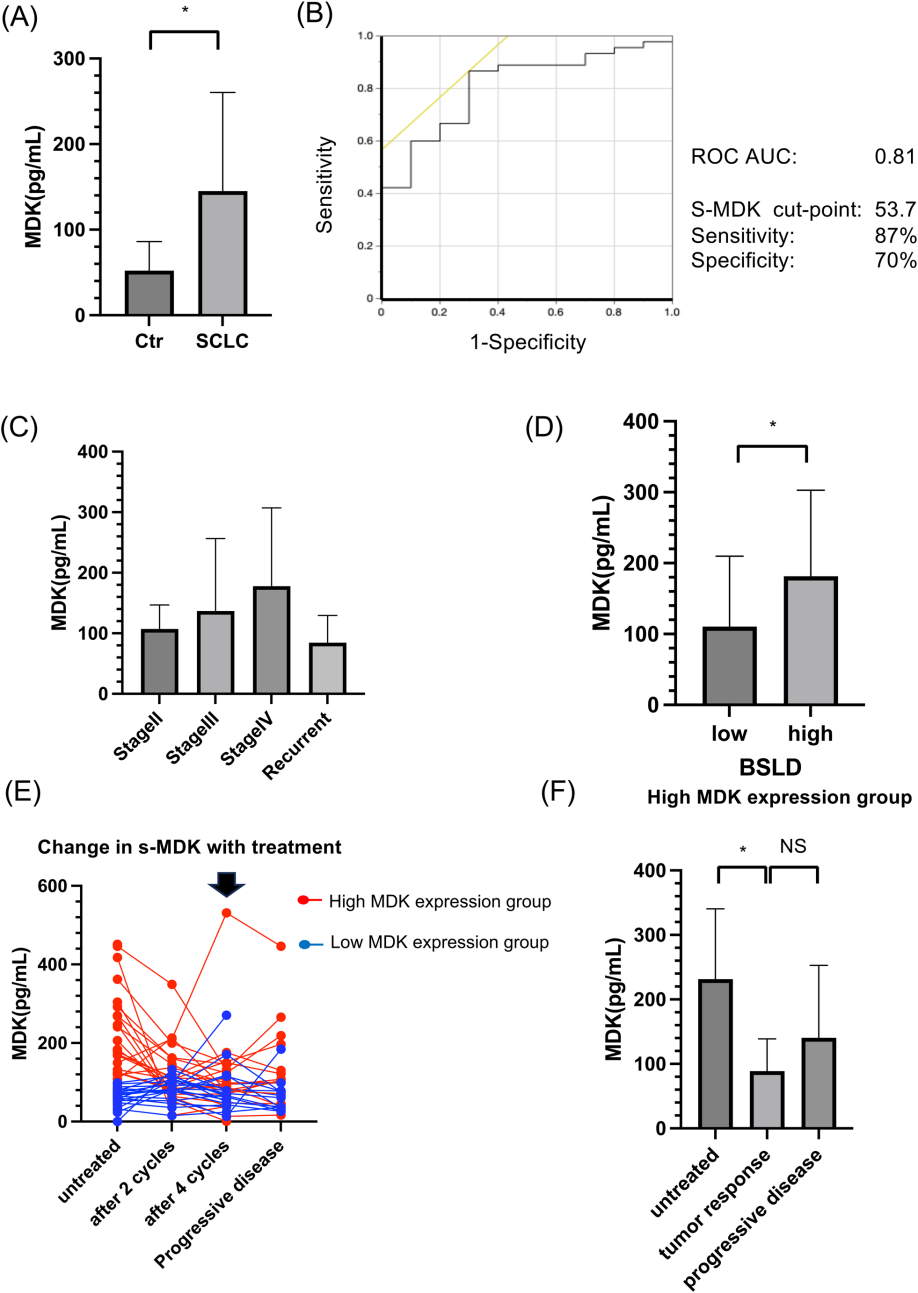
Human s‐MDK concentrations generally reflect the SCLC tumor burden. (A) s‐MDK levels in patients with SCLC were measured using an ELISA kit before treatment and compared with those in healthy controls. (B) ROC curve and diagnostic value of s‐MDK as an SCLC tumor marker; AUC = 0.81. (C) s‐MDK levels in patients with SCLC at each tumor–node–metastasis stage. (D) Tumor burden of patients with SCLC evaluated using the BSLD of the target lesion. (E) Changes in s‐MDK levels during treatment. The arrow indicates the case in which s‐MDK levels increased rapidly during treatment and the tumor progressed immediately after four cycles of chemotherapy (Figure S1A). (F) s‐MDK levels before treatment, during tumor response, and during tumor progression in the high‐MDK expression groups. *p < 0.05; NS, *p* > 0.05. AUC, area under the curve; BSLD, baseline sum of the longest diameter; NS, not significant; ROC, receiver operating characteristic; SCLC, small cell lung cancer; s‐MDK, serum midkine.

We measured s‐MDK levels in 45 treatment‐naive patients with SCLC and 10 healthy controls using ELISA. The profiles of these patients are summarized in Table 1. Except for treatment, no significant difference in patient characteristics was observed between the high‐ and low‐MDK expression groups. Patients with SCLC exhibited significantly higher s‐MDK levels than healthy controls (*p* < 0.05) (Figure 1A). Based on the ROC curve analysis, an AUC value of 0.81 was obtained (Figure 1B). s‐MDK levels increased with advancement in cancer stage (Figure 1C) and significantly increased in patients with high BSLD (Figure 1D). In the high‐MDK expression group, s‐MDK levels significantly decreased following tumor response and increased during tumor progression (Figure 1E,F). In a patient who exhibited markedly increased s‐MDK levels after four cycles of chemotherapy, the tumor progressed immediately after chemotherapy, although no increase in the expression of pro‐gastrin‐releasing peptide, a representative SCLC marker, was observed (Figure S1A). A trend toward better prognosis was observed in the low‐MDK expression group as compared to the high‐MDK expression group (Figure S1B,C).

**TABLE 1:** Patient characteristics with respect to s‐MDK measurements.

	All (*n* = 45)	MDK‐high (*n* = 22)	MDK‐low (*n* = 23)	*p*
Age (years)				
Median (range)	70 (46–80)	70.5 (48–79)	69 (46–80)	0.6956
Smoking status				0.3977
Never smoked	0	0	0	
Former smoker	30	16	14	
Current smoker	15	6	9	
Stage				0.8908
Stage II	5	2	3	
Stage III	18	8	10	
Stage IV	18	10	8	
Recurrence	4	2	2	
Treatment				0.0015
Chemotherapy	17	7	10	
Chemotherapy + ICI	16	13	3	
Chemotherapy + radiation	12	2	10	

Abbreviations: MDK‐high, high‐MDK expression group; MDK‐low, low MDK expression group.

### 3.2 MDK Expression in SCLC Is Variable and Has no Clear Correlation With Subgroups or NE Features

**FIGURE 2:**
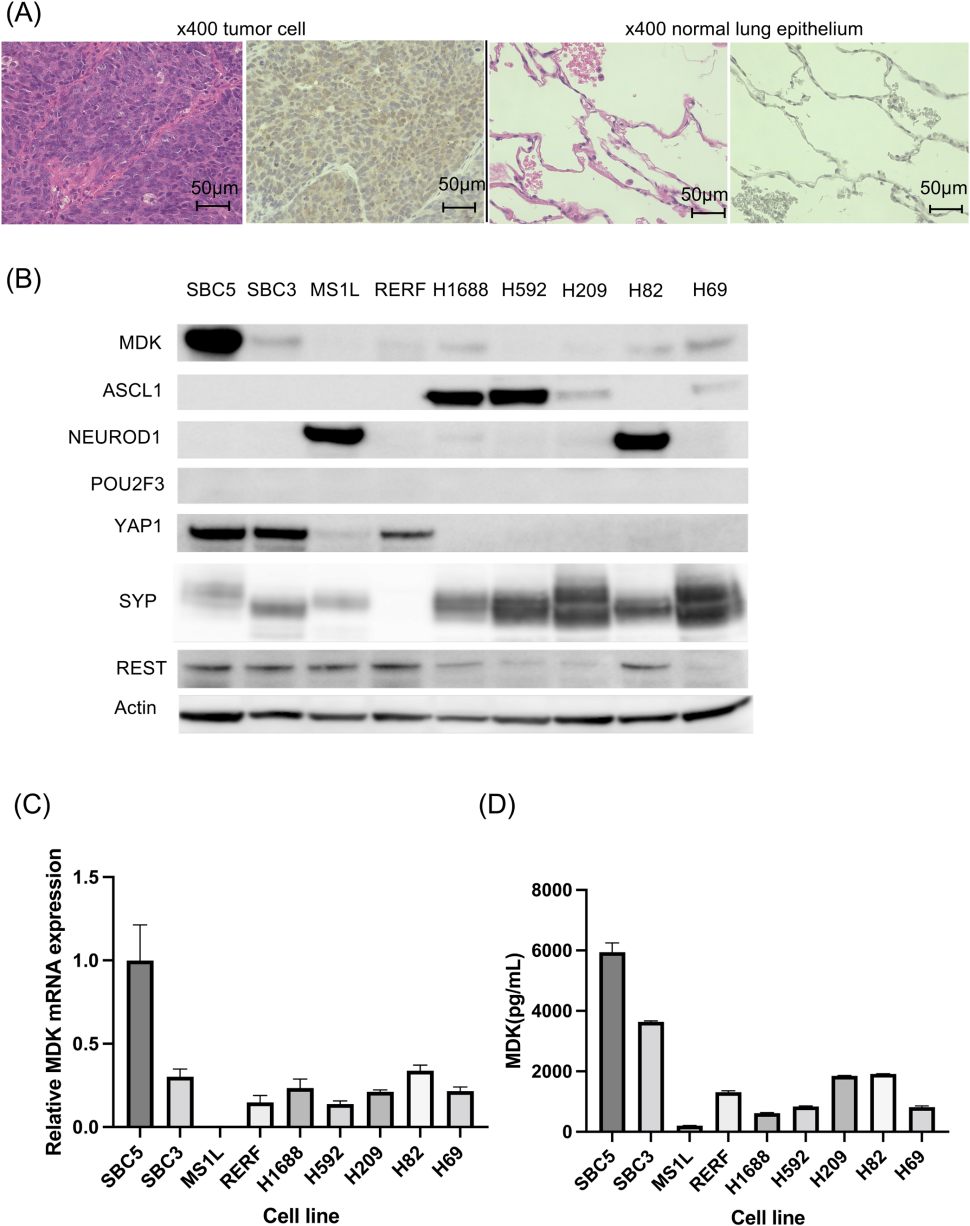
MDK expression in small cell lung cancer (SCLC) cells is variable and has no clear correlation with subgroups or neuroendocrine (NE) features. (A) MDK expression in human SCLC tissues as assessed through immunohistochemistry. Representative images of cancerous and normal lung tissues in the same specimen are shown. (B) Protein levels of MDK, SCLC subtype markers, the NE marker, SYP, and the non‐NE marker, REST, in SCLC cell lines as determined through western blotting. (C) MDK mRNA expression in SCLC cell lines as evaluated through qRT‐PCR. (D) MDK secretion in SCLC cell lines in conditioned medium as determined using ELISA. ASCL1, Achaete‐scute family bHLH transcription factor 1; MDK, Midkine; NEUROD1, neuronal differentiation 1; POU2F3, POU class 2 homeobox 3; RERF, RERF‐LC‐MA cells; REST, RE1 silencing transcription factor; SYP, Synaptophysin; YAP1, Yes‐associated protein 1.

Next, we evaluated MDK expression in surgically resected human SCLC tumors. Of note, MDK was detected in the cytoplasm after staining, but was not observed in normal lung tissues (Figure 2A). Moreover, the expression of MDK, SCLC transcriptional subtype markers, the NE marker, SYP, and the non‐NE marker, REST, was evaluated through IHC using four surgically resected human SCLC sections. Immunostaining images are shown in Figure S2, and the profiles of these patients are summarized in Table 2. Upregulated and extensive MDK expression was observed in Case 2, whereas in the other cases, it was expressed only in some parts of the tumor. No correlation was observed between the expression of MDK and that of SCLC subtype markers or NE markers in the four cases. Next, we determined MDK protein and mRNA levels in the nine human SCLC cell lines using western blotting and qRT‐PCR, respectively (Figure 2B,C). Furthermore, MDK secretion in the nine cell lines was measured using ELISA (Figure 2D). MDK was found to be most upregulated and secreted in the SBC5 cell line. It was moderately or weakly expressed and secreted in all cell lines except MS1L (Figure 2B–D). Further, the expression of the SCLC subtype markers, SYP and REST, in each cell line was evaluated through western blotting (Figure 2B). Based on the results depicted in Figure 2B–D, MDK protein and mRNA expression, as well as its secretion, in each SCLC molecular subtype (defined by ASCL1/NEUROD1/POU2F3/YAP1 expression) was evaluated (Figure S3A–C). There were no significant differences in MDK expression between SCLC subgroups. In addition, correlations between MDK expression/secretion and NE features were assessed using simple linear regression analyses (Figure S3D–I). No correlation was observed between MDK expression/secretion and NE features. Furthermore, although the number of surgical specimens was small (only four cases) and only included as a reference finding, no match was found between cell lines and surgical specimens. Based on these findings, we concluded that there was no correlation between MDK expression and SCLC subgroup or NE features.

**TABLE 2:** Characteristics of patients evaluated using immunohistochemistry.

	Case 1	Case 2	Case 3	Case 4
Age^a^ (year)	71	67	73	76
Sex	Male	Male	Male	Male
Smoking history	Former smoker	Former smoker	Former smoker	Former smoker
Brinkman Index	1080	550	600	1060
Clinical stage	cT1cN0M0	cT1bN0M0	cT1bN1M0	cT1bN0M0
Pathological stage	pT1c	pT1b	pT1cN1M0	pT1b
Pathological diagnosis	SCLC	SCLC	SCLC	SCLC
Surgical procedure	Limited resection	Limited resection	Lobectomy	Limited resection
Tumor site	RLL	RLL	LUL	LLL

Abbreviations: LLL, left lower lobe; LUL, left upper lobe; RLL, right lower lobe.

^a^ Age at time of surgery.

### 3.3 MDK Promotes Cell Proliferation and Migration in SCLC

**FIGURE 3:**
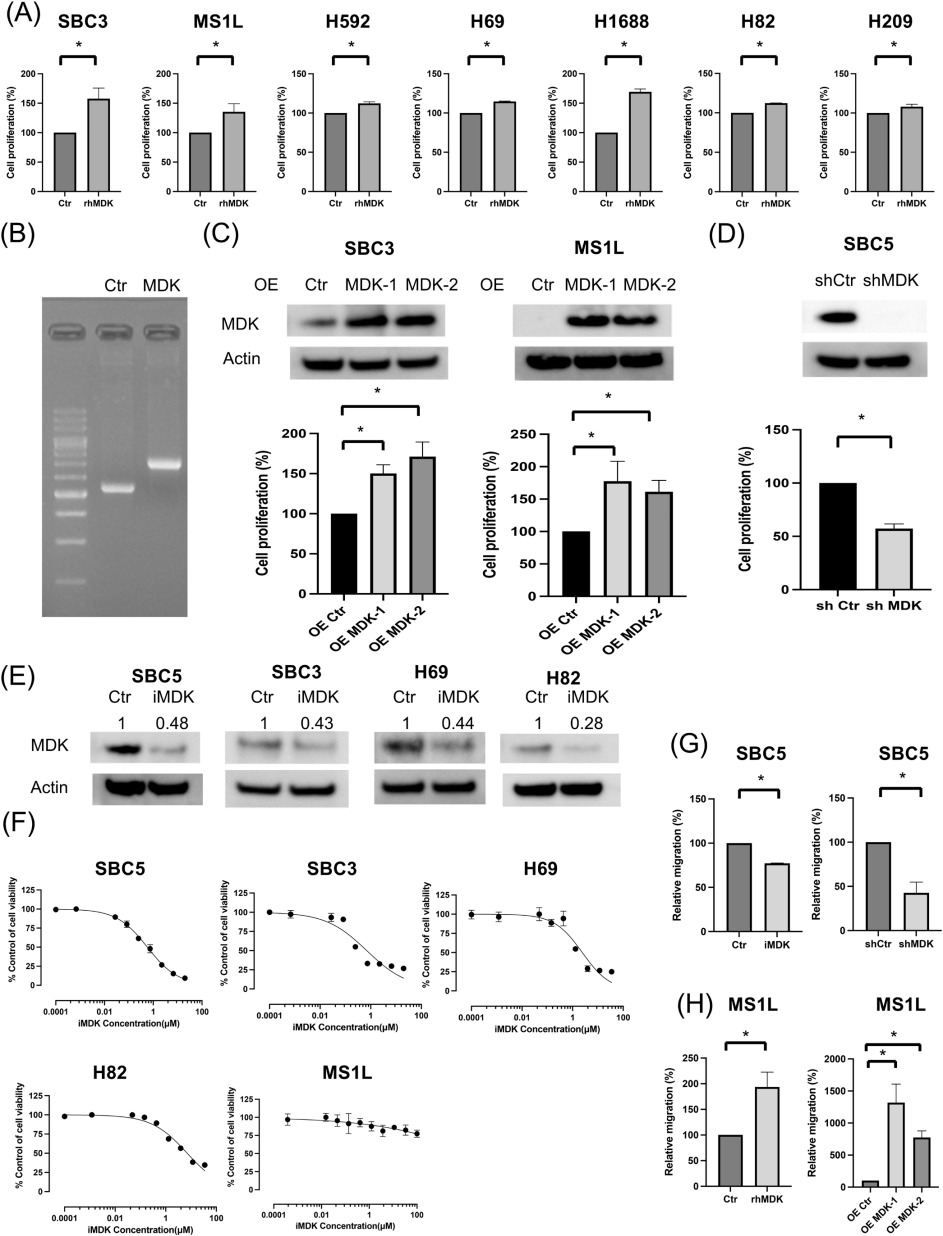
MDK regulates small cell lung cancer (SCLC) cell proliferation and migration. (A) Cell growth was measured using MTT assays 96 h after cell seeding with rhMDK (100 ng/mL) (*n* = 3; mean ± standard deviation [SD]). (B) Gel electrophoresis was used to determine whether the plasmids made from RG221818 and PS100019 retained the target genes. (C) Cell growth was measured using MTT assays 96 h after cell seeding for SBC3 and MS1L cells transfected with a control vector or an MDK‐expression vector (*n* = 3; mean ± SD) and (D) for SBC5 cells transfected with shCtr or shMDK (*n* = 3; mean ± SD). (E) MDK expression suppression (after 48 h) upon iMDK administration as evaluated through western blotting. (F) Representative cell viability data for SCLC cell lines treated with iMDK for 72 h as assessed through the MTT assay (*n* = 3; mean ± SD). (G) Migration results for SBC5 cells treated with iMDK for 24 h or subjected to MDK expression suppression using shMDK; the number of migrated cells was determined in five random fields of view (*n* = 3; mean ± SD). (H) Migration results for MS1L cells treated with rhMDK (100 ng/mL) or MS1L cells overexpressing MDK; after incubation for 48 h, the number of migrated cells was determined in five random fields of view (*n* = 3; mean ± SD). *p < 0.05. Ctr, Control; iMDK, MDK inhibitor; MDK, Midkine; OE, overexpression; rhMDK, recombinant human MDK; shCtr, control shRNA vector; shMDK, ShRNA against MDK.

Analysis of the effect of MDK on cell proliferative and migratory capacity in SCLC showed that rhMDK administration promoted proliferation in SCLC cells with no or low MDK expression (Figure 3A). Additionally, MDK overexpression induced using the MDK expression vector promoted cell proliferation in the SBC3 and MS1L cell lines (Figure 3C). In contrast, suppression of MDK expression in the SBC5 cell line using shMDK impaired tumor cell proliferation (Figure 3D). iMDK inhibited MDK expression (Figure 3E) and exerted concentration‐dependent cytotoxic effects on MDK‐expressing SCLC cells, but not on non‐MDK‐expressing MS1L cells (Figure 3F, Table S3). Furthermore, suppression of MDK expression using iMDK and shMDK decreased migration capacity in SBC5 cells, whereas rhMDK administration and MDK overexpression improved this capacity in MS1L cells (Figure 3G,H). Representative images for migration assay findings are shown in Figure S4.

### 3.4 MDK Activates Intracellular Signaling Pathways Mainly Through the AKT Pathway

**FIGURE 4:**
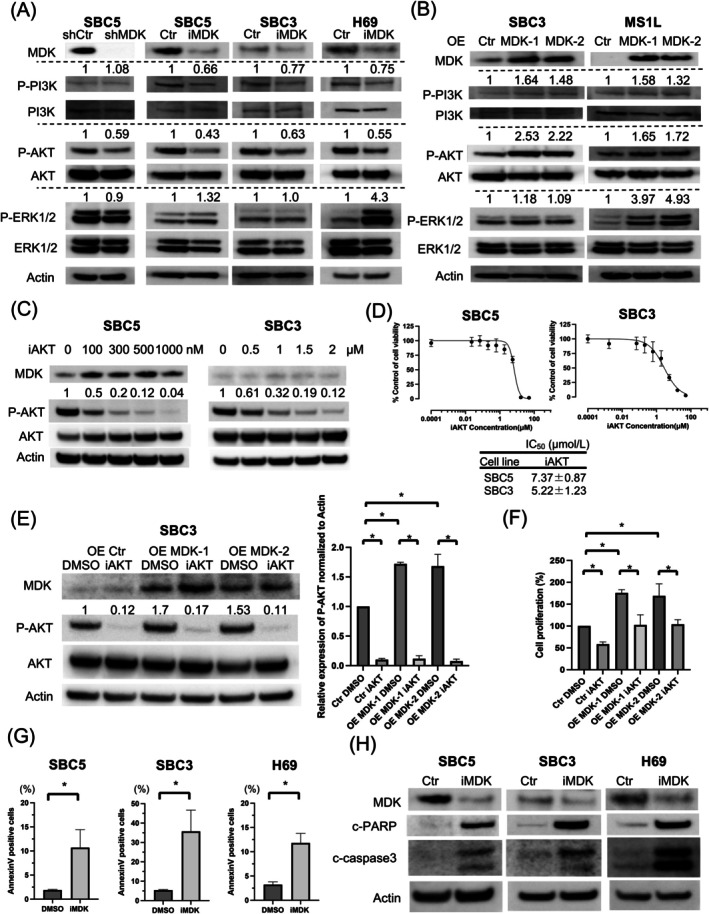
MDK upregulates the PI3K/AKT pathway and inhibits small cell lung cancer cell apoptosis. (A) P‐PI3K, P‐AKT, and P‐ERK protein levels upon MDK knockdown in SBC5, SBC3, and H69 cells as determined through western blotting. (B) P‐PI3K, P‐AKT, and P‐ERK protein levels upon MDK overexpression in SBC3 and MS1L cells as determined through western blotting. (C) P‐AKT expression suppression (after 48 h) upon iAKT administration as evaluated through western blotting. (D) Representative cell viability data for SBC5 and SBC3 cells treated with iAKT for 72 h as determined through the MTT assay (*n* = 3; mean ± standard deviation [SD]). (E) iAKT induced P‐AKT downregulation in both SBC3 control and MDK‐overexpressing cells. (F) iAKT administration suppressed the MDK overexpression‐induced enhancement of cell proliferation. (G) Flow cytometric analysis results with Annexin V/propidium iodide staining showing apoptotic cells after treatment with iMDK in SBC5, SBC3, and H69 cells. The bar graphs show the summary data for the percentage of Annexin V‐positive cells (*n* = 3; mean ± SD). (H) c‐PARP and c‐caspase 3 levels in cells treated with iMDK as determined through western blotting 48 h after drug administration. *p < 0.05. AKT, protein kinase B; c, cleaved; Ctr, control; DMSO, dimethyl sulfoxide; ERK, extracellular signal‐regulated kinase; iAKT, AKT inhibitor; iMDK, MDK inhibitor; MDK, Midkine; OE, overexpression; P, phosphorylated; PARP, poly‐ADP ribose polymerase; PI3K, phosphatidylinositol 3‐kinase; shCtr, control shRNA vector; shMDK, ShRNA against MDK.

As MDK activates the PI3K/AKT and MAPK pathways and contributes to cell survival [17], we evaluated its effects on these signaling pathways. Suppression of MDK expression in SBC5 cells using shMDK downregulated P‐AKT expression. Moreover, iMDK downregulated P‐AKT expression in three cell lines (Figure 4A). Although shMDK did not alter P‐PI3K expression in SBC5 cells, iMDK downregulated its expression in three cell lines (Figure 4A). MDK overexpression upregulated P‐PI3K and P‐AKT expression in SBC3 and MS1L cells (Figure 4B). Conversely, MDK suppression did not alter P‐ERK expression in SBC5 and SBC3 cells, but upregulated its expression in H69 cells (Figure 4A). In addition, MDK overexpression did not alter P‐ERK expression in SBC3 cells but upregulated it in MS1L cells (Figure 4B). Findings for the quantification and comparison of change in P‐AKT and P‐ERK expression based on the adjustment of MDK expression in Figure 4A,B are presented in Figure S5. Of note, iAKT administration inhibited cell proliferation in the SBC5 and SBC3 cell lines (Figure 4C,D). Furthermore, to confirm the correlation between MDK expression and the AKT pathway, we evaluated whether cell proliferation, which is promoted following MDK overexpression, is suppressed by iAKT administration. P‐AKT expression was downregulated following iAKT administration in both control and MDK‐overexpressing cells (Figure 4E). iAKT suppressed the MDK overexpression‐induced increase in cell proliferation (Figure 4F). Additionally, iMDK significantly induced apoptosis (Figure 4G) and increased cleaved (c)‐poly‐ADP ribose polymerase and c‐caspase 3 levels (Figure 4H). The Notch pathway plays an important role in SCLC tumor heterogeneity [13], and Notch2, an MDK receptor candidate, is an airway neuroendocrine stem cell marker [34]. Therefore, we evaluated the expression of Notch pathway‐related proteins in SCLC cells. All MDK‐expressing SCLC cell lines, except H209 and H69, exhibited positive expression for the Notch2 intracellular domain (Figure 2B–D, Figure S6A). Furthermore, MDK knockdown in SBC5, the SCLC cell line with the highest MDK expression and Notch pathway activation, resulted in the suppression of the Notch pathway (Figure S6B). In contrast, MDK overexpression in SBC3 and MS1L cells did not induce activation of the Notch pathway, nor Notch2 expression (Figure S6C). These findings indicate that MDK is associated with multiple intracellular pathways in SCLC. More specifically, the AKT pathway exhibited a significant correlation with MDK expression in all cell lines evaluated in this study, suggesting that it is a major intracellular pathway regulated by MDK.

### 3.5 MDK Attenuated the Antitumor Effect of CDDP in SCLC Cells

**FIGURE 5:**
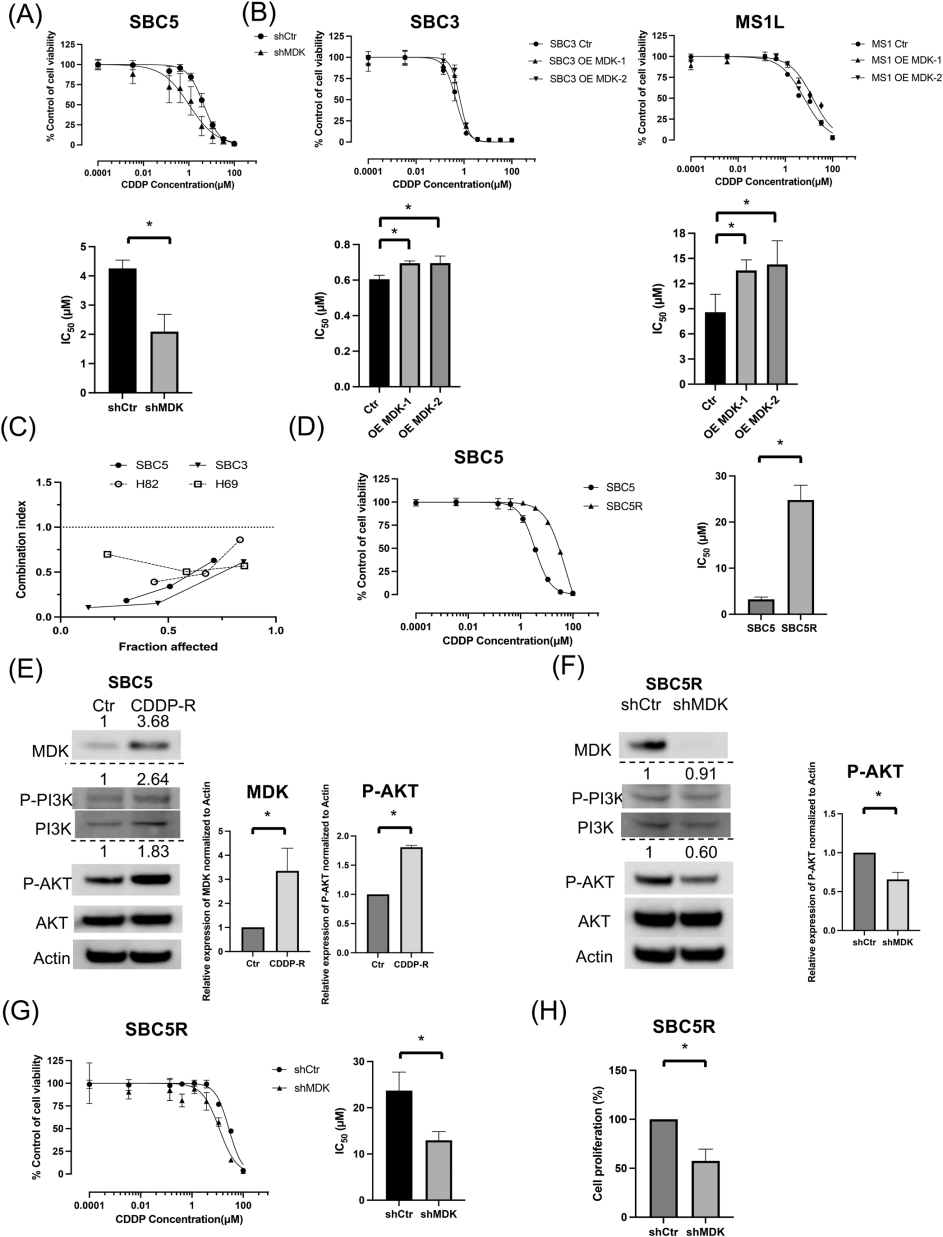
MDK attenuates the effects of CDDP and may be associated with CDDP resistance in small cell lung cancer (SCLC). (A) Changes in the effects of CDDP in SBC5 cells treated with CDDP for 72 h upon MDK knockdown as determined through the MTT assay (*n* = 3; mean ± standard deviation [SD]). (B) Changes in the effects of CDDP in SBC3 and MS1L cells treated with CDDP for 72 h upon MDK overexpression as determined through the MTT assay (*n* = 3; mean ± SD). (C) Effects of combined treatment with CDDP and iMDK for 72 h in SCLC cell lines as determined using the combination index. (D) MTT assay for the CDDP‐R SCLC cell lines, SBC5 and SBC5R (*n* = 3; mean ± SD). (E) MDK expression and PI3K/AKT pathway activity in SBC5 and SBC5R cells as determined through western blotting. (F) Changes in MDK expression and PI3K/AKT pathway activity in SBC5R cells upon MDK expression suppression using shMDK as evaluated through western blotting. (G) Changes in the effects of CDDP in SBC5R cells treated with CDDP for 72 h upon MDK knockdown as determined through the MTT assay (*n* = 3; mean ± SD). (H) Cell growth at 96 h in SBC5R cells transfected with shCtr or shMDK after cell seeding as evaluated through MTT assays (*n* = 3; mean ± SD). *p < 0.05; NS: *P* > 0.05. AKT, protein kinase B; CDDP, cisplatin; CDDP‐R, cisplatin resistant; Ctr, control; iMDK, MDK inhibitor; MDK, Midkine; NS, not significant; OE, overexpression; P, phosphorylated; PI3K, phosphatidylinositol 3‐kinase; shCtr, control shRNA vector; shMDK, shRNA against MDK.

The AKT pathway can induce resistance to CDDP [35]; hence, we investigated the effect of MDK on the antitumor effects of CDDP in SCLC cells. Of note, shMDK‐mediated MDK suppression in SBC5 cells enhanced the antitumor effects of CDDP (Figure 5A, Table S4). Conversely, MDK overexpression in SBC3 and MS1L cells attenuated the effects of CDDP (Figure 5B, Table S4). Additionally, a synergistic effect was observed between CDDP and iMDK in SCLC cell lines with MDK expression (Figure 5C). Next, we established the CDDP‐resistant cell lines, SBC5R and SBC3R (Figure 5D, Figure S7A, Table S3), which exhibited a more significant upregulation in MDK expression and more enhanced PI3K/AKT pathway activation than their parent cell lines, SBC5 and SBC3 (Figure 5E, Figure S7B). MDK knockdown in CDDP‐resistant cell lines using shMDK suppressed the AKT pathway and enhanced the antitumor effects of CDDP (Figure 5F,G, Figure S7C,D, Table S4). Furthermore, MDK suppression in SBC5R and SBC3R cells using shMDK impaired tumor cell proliferation (Figure 5H, Figure S7E). These results suggest that MDK might be involved in CDDP resistance in SCLC.

### 3.6 MDK Suppression Inhibits Tumor Growth in SCLC and Enhances the Effects of CDDP in Vivo

**FIGURE 6:**
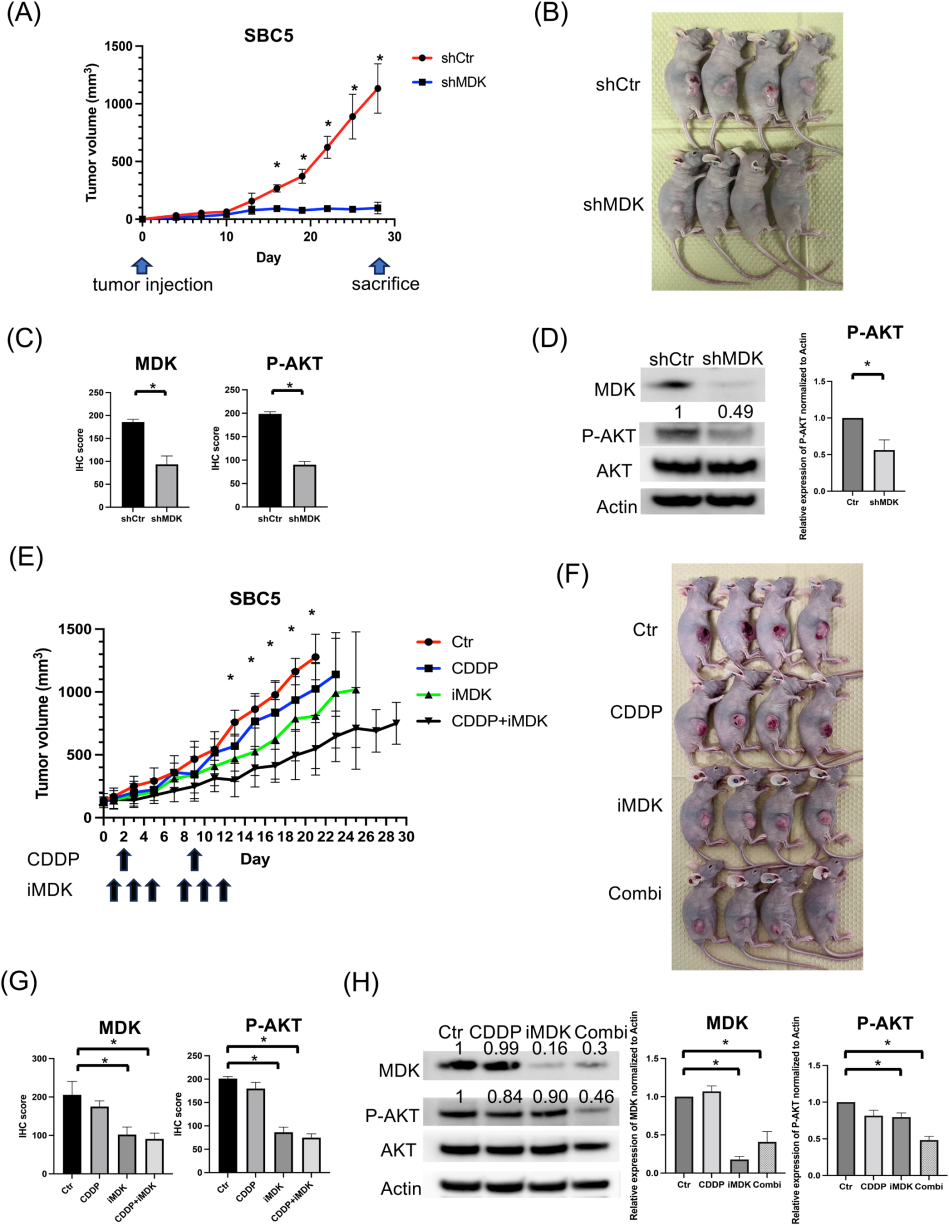
MDK suppression inhibits small cell lung cancer cell proliferation and enhances the effects of CDDP in vivo. (A) Tumor volume measurement in nude mice implanted with SBC5 cells transfected with shCtr or shMDK. Data indicate the average tumor volume (*n* = 4/group; mean ± standard deviation [SD]). (B) Photograph of tumors 28 days after implantation of SBC5 cells transfected with shCtr or shMDK. (C) Xenograft tumors from each group were removed 28 days after tumor induction. MDK and P‐AKT expression were evaluated based on IHC scores using an IHC assay. Representative HE and IHC images are shown in Figure S8A. (D) MDK and P‐AKT expression were evaluated through western blotting. (E) Tumor volume measurement in nude mice implanted with SBC5 cells; CDDP and iMDK were intraperitoneally injected once (1 mg/kg/day) and thrice (9 mg/kg/day) a week, respectively. The arrows show the timing for drug administration. Data indicate the average tumor volume (*n* = 4/group; mean ± SD). (F) Photograph of tumors at the end of the 2‐week treatment period. (G) Two days after drug administration, xenograft tumors from each treatment group were removed. MDK and P‐AKT expression was evaluated based on IHC scores using an IHC assay. Representative HE and IHC images are shown in Figure S8B. (H) MDK and P‐AKT expression were evaluated through western blotting. *p < 0.05. CDDP, cisplatin; Ctr, control; HE, hematoxylin and eosin; IHC, Immunohistochemistry; iMDK, MDK inhibitor; MDK, Midkine; shCtr, control shRNA vector; shMDK, ShRNA against MDK.

We evaluated the effect of MDK suppression on tumor growth in vivo. The tumor burden in nude mice implanted with shMDK SBC5 cells was significantly lower than that in control mice (Figure 6A,B). Decreased MDK protein levels were maintained in resected tumors using shMDK at day 28 following tumor induction (Figure 6C,D, Figure S8A). Furthermore, P‐AKT expression was also downregulated in shMDK‐treated tumors (Figure 6C,D, Figure S8A). The iMDK and CDDP combination inhibited tumor progression numerically compared to monotherapy (Figure 6E,F). No significant weight loss was observed in mice during the treatment (data not shown). Additionally, MDK and P‐AKT expression was suppressed in resected tumors following iMDK administration with or without CDDP (Figure 6G,H, Figure S8B).

## 4 Discussion

In this study, we demonstrated that serum MDK levels increase in patients with SCLC, and that MDK regulates cell proliferation and the antitumor effects of CDDP in vitro and in vivo. To the best of our knowledge, ours is the first study to evaluate MDK expression and function in SCLC.

Several clinical trials have been carried out on targeted therapies for SCLC and several others are ongoing; however, the data reported are limited [36]. SCLC is heterogeneous, with several SCLC subtypes existing simultaneously within a tumor, indicating that comprehensive treatment, and not just a single treatment, is necessary for its management [7, 12, 13, 37]. Lim et al. suggested that non‐NE‐type SCLC cells secrete MDK, which promotes NE‐type SCLC cell growth [12]. Thus, we hypothesized that MDK‐targeted therapy might be efficacious in treating tumors with various SCLC cells as MDK may target non‐NE‐type SCLC cells, thereby indirectly inhibiting NE‐type SCLC cell proliferation. Although our results did not show a correlation between SCLC subtype or NE features and MDK expression in human tumor tissues and SCLC cell lines, MDK was expressed in all subtypes, with both non‐NE‐ and NE‐type SCLC cells being sensitive to MDK inhibition‐based treatments. These results suggest that MDK may be a versatile therapeutic target for SCLC. However, further studies are required to clarify the association between MDK expression and SCLC characteristics.

Upregulated MDK expression in tumor tissues is associated with tumor growth in many cancers, including non‐SCLC (NSCLC), hepatocellular carcinoma, and bladder cancer [20, 27, 28]. Furthermore, as compared to healthy controls, patients with several cancers have been shown to exhibit upregulated s‐MDK expression, which is useful as a noninvasive parameter for assessing disease diagnosis and progression [20, 38, 39, 40, 41, 42]. In this study, for the first time, we found increased s‐MDK expression levels in patients with SCLC and observed a correlation between increased s‐MDK levels and SCLC tumor burden. SCLC is mostly diagnosed at an advanced stage, and owing to its high malignant potential, prompt treatment is often required. Thus, determining s‐MDK levels could help in the clinical diagnosis of SCLC. Furthermore, we found that s‐MDK levels decreased following treatment in patients with high s‐MDK secretion; in one case (Figure S1A), increased s‐MDK levels were detected before disease progression as determined based on imaging and the analysis of tumor markers, indicating that it could serve as a predictive factor for disease progression during treatment.

MDK mediates cancer cell proliferation, survival, migration, and chemotherapeutic resistance via the PI3K/AKT and MAPK pathways [30, 43, 44]. In this study, we found that MDK activated the AKT pathway in SCLC cells; however, its correlation with the MAPK pathway was unclear. Hao et al. reported that MDK inhibition using iMDK induced only suppression of the PI3K/AKT pathway in NSCLC; however, it is unclear why the MAPK pathway was not inhibited [28]. We found P‐ERK to be upregulated in H69 cells following MDK suppression, and this may have been compensated for by the downregulation of the PI3K/AKT pathway. In MS1L cells, both the PI3K/AKT and MAPK pathways were enhanced following MDK overexpression, indicating that pathway inhibition or activation might be cell‐context‐dependent.

We found that iMDK and CDDP exerted combined antitumor effects in vitro and in vivo. Moreover, MDK and the PI3K/AKT pathway were upregulated in the CDDP‐resistant SCLC cell lines. CDDP is one of the chemotherapeutic drugs currently used for the management of SCLC; however, acquired resistance to this drug affects treatment efficacy. MDK is overexpressed in several cancers and mediates resistance to chemotherapeutic drugs via several mechanisms [30]. It renders glioma cells resistant to tetrahydrocannabinol and inhibits autophagy‐mediated cell death activation via the AKTmTORC1 pathway [45]. In gastric cancer, MDK overexpression upregulates P‐AKT and P‐ERK expression, thereby inducing resistance to adriamycin [46]. Ying et al. reported that PI3K/AKT pathway activation is associated with chemotherapeutic resistance in SCLC [35], indicating that PI3K/AKT pathway suppression might be the main reason why MDK inhibition enhanced the efficacy of CDDP in this study.

Our study had several limitations. First, owing to the small number of IHC evaluations and SCLC cell lines, there is insufficient data to confidently conclude that no correlation exists between MDK expression and SCLC subtypes. Second, the ages of patients in the control group for s‐MDK measurement were relatively lower than those of patients with SCLC. It has been reported that s‐MDK expression gradually decreases with age in healthy children; hence, the difference in age may have affected our results [47]. Third, owing to the small sample size and variable disease stages of patients for which MDK levels were measured, it was difficult to evaluate the correlation between s‐MDK and prognosis. Lastly, although the use of ICIs in combination with chemotherapy is one of the standard therapeutic approaches for SCLC management, we did not evaluate the effect of MDK on the effects of ICIs. MDK was found to suppress CD8 T‐cell function by stimulating immunosuppressive myeloid‐derived suppressor cells, partly driven by nuclear factor‐κB in the TME, resulting in resistance to ICIs [25, 26]. If MDK inhibition improves T‐cell function and the TME in SCLC, combining iMDK with ICIs and platinum‐based chemotherapy may represent a promising therapeutic approach.

Collectively, ours is the first study to evaluate MDK expression and function in SCLC, and the finding that MDK inhibition enhances CDDP efficacy is highly significant as it presents MDK as a potential therapeutic target for SCLC. These findings reveal the pivotal role played by MDK in SCLC, suggesting that it is a potential therapeutic target for SCLC.

## Author Contributions

**Shotaro Ito:** writing – original draft, writing – review and editing, investigation, conceptualization, methodology, data curation, validation, formal analysis, visualization, project administration. **Jun Sakakibara‐Konishi:** conceptualization, methodology, data curation, supervision, writing – review and editing, validation, formal analysis, project administration. **Mineyoshi Sato:** writing – review and editing. **Tetsuaki Shoji:** writing – review and editing. **Megumi Furuta:** writing – review and editing. **Hirofumi Takahashi:** writing – review and editing. **Kosuke Tsuji:** writing – review and editing. **Daisuke Morinaga:** writing – review and editing. **Masahiro Kashima:** writing – review and editing. **Hidenori Kitai:** writing – review and editing. **Junko Kikuchi:** writing – review and editing. **Eiki Kikuchi:** writing – review and editing. **Kanako C. Hatanaka:** writing – review and editing. **Yutaka Hatanaka:** writing – review and editing. **Kyoko Hida:** writing – review and editing. **Takuro Noguchi:** writing – review and editing. **Satoshi Konno:** writing – review and editing.

## Ethics Statement

This study was performed in line with the principles of the Declaration of Helsinki. Approval was granted by the institutional review board of Hokkaido University (identification number: 020–0379). All animal experiments were performed according to protocols approved by the Institutional Animal Care Committee of Hokkaido University (approval number: 23–0091).

## Consent

Patient consent: Informed consent was obtained from all individual participants included in this study. Consent to publish: All individuals provided signed informed consent regarding the publishing of their data.

## Conflicts of Interest

The authors declare no conflicts of interest.

## Supporting information


Data S1.

Figures S1‐S8.

Tables S1‐S4.

## Data Availability

The data that support the findings of this study are available on request from the corresponding author. The data are not publicly available due to privacy or ethical restrictions.
