# Supplementary material for: Midkine Promotes Tumor Growth and Attenuates the Effect of Cisplatin in Small Cell Lung Cancer
Source: Cancer Med. 2025 Jul 7;14(13):e71034. doi: 10.1002/cam4.71034 (PMC12230509; doi:10.1002/cam4.71034)
Supplement: Supplementary file 1 — Data S1. [file CAM4-14-e71034-s003.docx]

Cancer Medicine

Midkine promotes tumor growth and attenuates the effect of cisplatin in small cell lung cancer

Shotaro Ito^1^, Jun Sakakibara-Konishi^1^, Megumi Furuta^1^, Tetsuaki Shoji^1^, Hirofumi Takahashi^1^, Mineyoshi Sato^1^, Kosuke Tsuji^1^, Daisuke Morinaga^1^, Masahiro Kashima^1^, Hidenori Kitai^1^, Junko Kikuchi^1^, Eiki Kikuchi^1^, Kanako C Hatanaka^2^, Yutaka Hatanaka^2^, Kyoko Hida^3^, Takuro Noguchi^4^ and Satoshi Konno^1^

*^1^Department of Respiratory Medicine, Faculty of Medicine, Hokkaido University, Sapporo, Japan*

*^2^Center for Development of Advanced Diagnostics, Hokkaido University Hospital, Sapporo, Japan*

*^3^Department of Vascular Biology and Molecular Pathology, Hokkaido University Faculty of Dental Medicine, Sapporo, Japan*

*^4^Department of Medical Oncology, Faculty of Medicine and Graduate School of Medicine, Hokkaido University, Sapporo, Japan*

*****Corresponding author: Jun Sakakibara-Konishi, MD, PhD

Department of Respiratory Medicine, Faculty of Medicine, Hokkaido University, N 15, W 7, Kita-ku, Sapporo, 0608638, Japan

E-mail: [konishj@med.hokudai.ac.jp](mailto:konishj@med.hokudai.ac.jp)

**Supplementary information**

**MDK knockdown using small hairpin RNA (shRNA) against MDK**

Plasmids encoding human MDK (TR319183, ORIGENE) and control shRNA (TR30012, ORIGENE) vectors were purchased from OriGene Technologies. SBC5 cells were divided into two equal groups i.e., the shMDK (transfected with MDK shRNA) and shCtr (transfected with a control shRNA vector) groups. The day before transfection, 2 × 10^5^ cells were seeded per well onto six-well plates, and then transfected with 4 μg of the MDK shRNA plasmid in serum-free Opti-MEM I and 12 μL of TurboFectin 8.0. After 24 h, the transfected cells were diluted at a 1:10 ratio in 10-cm dishes, and the culture medium was replaced with complete medium containing puromycin (Sigma-Aldrich). The culture was screened using puromycin to obtain stable and positive clones.

**Migration assay**

Cell migration assays were performed using 24-well transwell plates. SBC5 and MS1 cells were plated onto the upper chamber which contained culture medium supplemented with 0.1% FBS; the lower chamber contained culture medium supplemented with 20% FBS. After incubation for 24 h (SBC5 cells) or 48 h (MS1 cells), the membranes were stained with Diff-Quik (Sysmex, Hyogo, Japan). The number of migrated or invaded cells was determined in five random fields of view using a BZ-9000 microscope (KEYENCE, Osaka, Japan).

**Apoptosis assay**

The apoptosis assay was performed using Annexin V, propidium iodide (PI), and the MEBCYTO Apoptosis Kit (Annexin V-FITC kit) (MBL, Nagoya, Japan) on the BD FACSVerse flow cytometer (Becton Dickinson, Franklin Lakes, NJ, USA) according to the instructions of the manufacturer. Annexin V-positive–PI-negative and Annexin V-positive–PI-positive cell populations exhibited early and late apoptosis, respectively.
