# Supplementary material for: Midkine Promotes Tumor Growth and Attenuates the Effect of Cisplatin in Small Cell Lung Cancer
Source: Cancer Med. 2025 Jul 7;14(13):e71034. doi: 10.1002/cam4.71034 (PMC12230509; doi:10.1002/cam4.71034)
Supplement: Supplementary file 2 — Figures S1‐S8. [file CAM4-14-e71034-s001.pdf]

Supplementary Figure 1

(A)

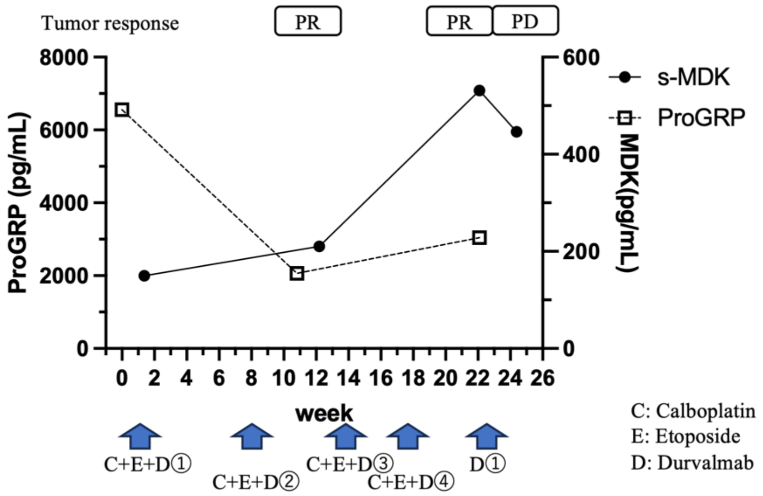

(B)

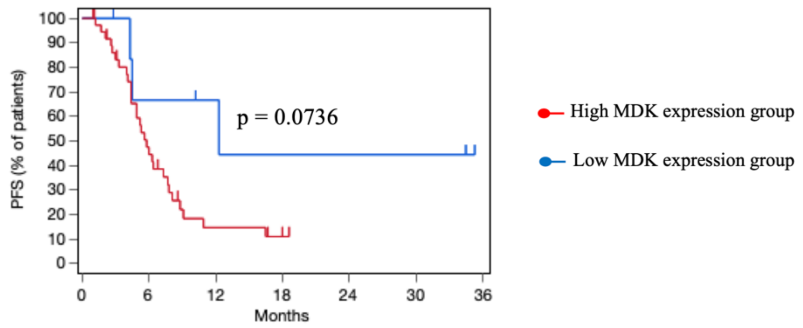

(C)

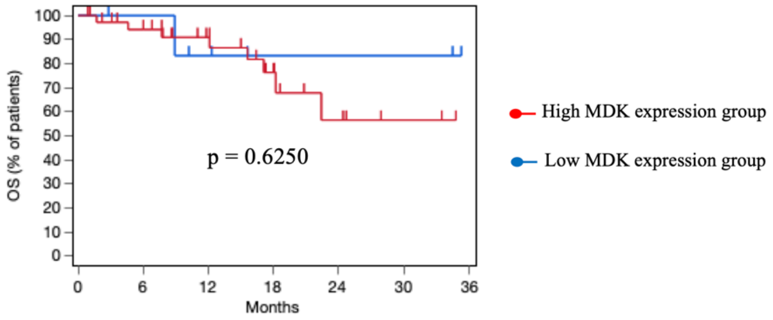

Supplementary Figure 1

MDK may serve as a tumor or prognostic biomarker for small cell lung cancer (SCLC)

(A) Clinical course of a patient who exhibited a rapid increase in s-MDK levels after four chemotherapy cycles and subsequent tumor progression. The left-side scale shows serum ProGRP levels, while the right-side scale shows s-MDK levels. Tumor response is shown above the graph. Arrows indicate anticancer drug administration. (B) Progression-free survival and (C) overall survival based on s-MDK levels. The high- and low-MDK expression groups were defined using the cut-off value optimized through an ROC analysis. SCLC: small cell lung cancer; s-MDK: serum MDK concentration; ProGRP: pro-gastrin-releasing peptide; PFS: progression-free survival; OS: overall survival

Supplementary Figure 2

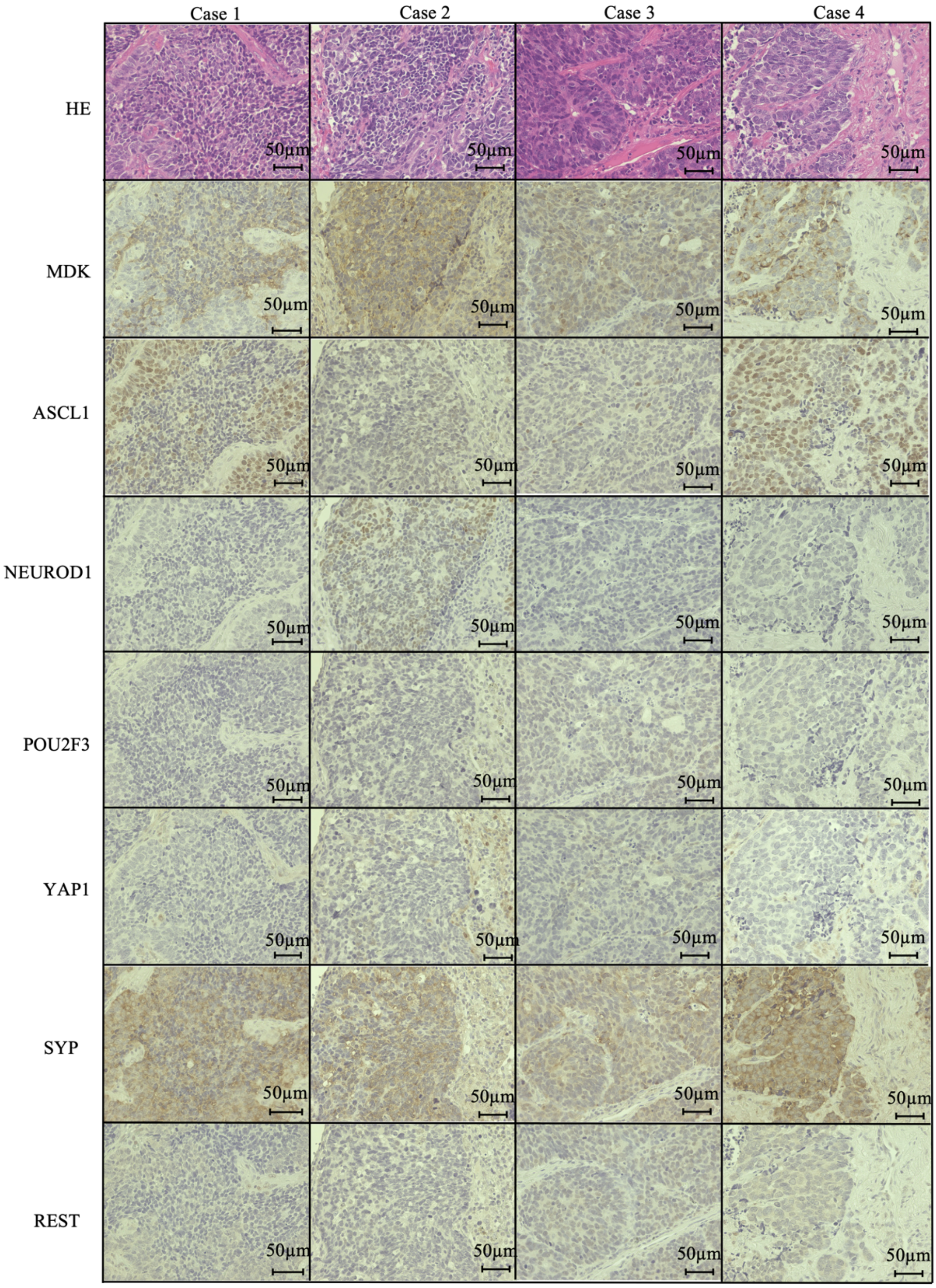

Supplementary Figure 2

The correlation between MDK expression and small cell lung cancer (SCLC) characteristics was evaluated through immunohistochemistry

MDK, ASCL1, NEUROD1, POU2F3, YAP1, SYP, and REST expression was immunohistochemically assessed in four surgical SCLC specimens. Representative high-magnification images for hematoxylin and eosin staining, as well as immunohistochemical staining, are shown.

SCLC: small cell lung cancer; SYP: synaptophysin; REST: RE1-silencing transcription factor

Supplementary Figure 3

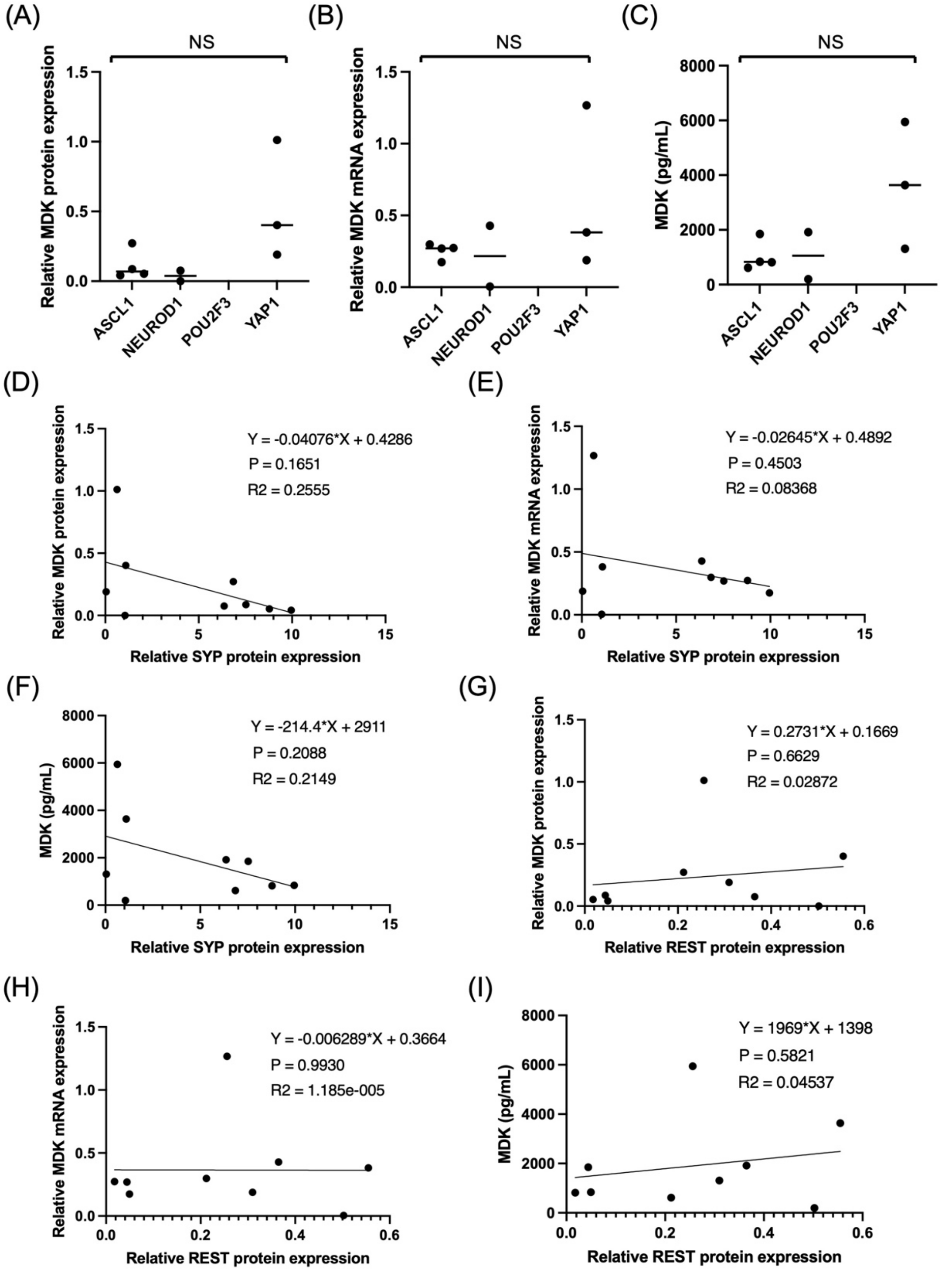

Supplementary Figure 3

Evaluation of the correlation between MDK expression and SCLC characteristics in nine SCLC cell lines. (A) MDK protein expression in SCLC cell lines is shown for each molecular subgroup. (B) MDK mRNA expression in SCLC cell lines is shown for each molecular subgroup. (C) MDK secretory capacity in SCLC cell lines is shown for each molecular subgroup. (D) The correlation between SYP expression and MDK protein expression in SCLC cell lines was evaluated using simple linear regression analyses. (E) The correlation between SYP expression and MDK mRNA expression in SCLC cell lines was evaluated using simple linear regression analyses. (F) The correlation between SYP expression and MDK secretory capacity in SCLC cell lines was evaluated using simple linear regression analyses. (G) The correlation between REST expression and MDK protein expression in SCLC cell lines was evaluated using simple linear regression analyses. (H) The correlation between REST expression and MDK mRNA expression in SCLC cell lines was evaluated using simple linear regression analyses. (I) The correlation between REST expression and MDK secretory capacity in SCLC cell lines was evaluated using simple linear regression analyses. MDK protein expression was assessed by western blotting, as shown in Figure 2B, using the image J software, and corrected for actin expression. MDK mRNA expression was evaluated through qRT-PCR as shown in Figure 2C. MDK secretory capacity was evaluated by ELISA using the concentration of MDK in the culture medium of each SCLC cell line as shown in Figure 2D. As shown in Figure 2B, SYP and REST protein expression was assessed by western blotting using the image J software, with correction

for actin expression. The Kruskal-Wallis test was used to determine significant differences between groups as shown in Supplementary Figure 3A-C. NS: $p > 0.05$

Supplementary Figure 4

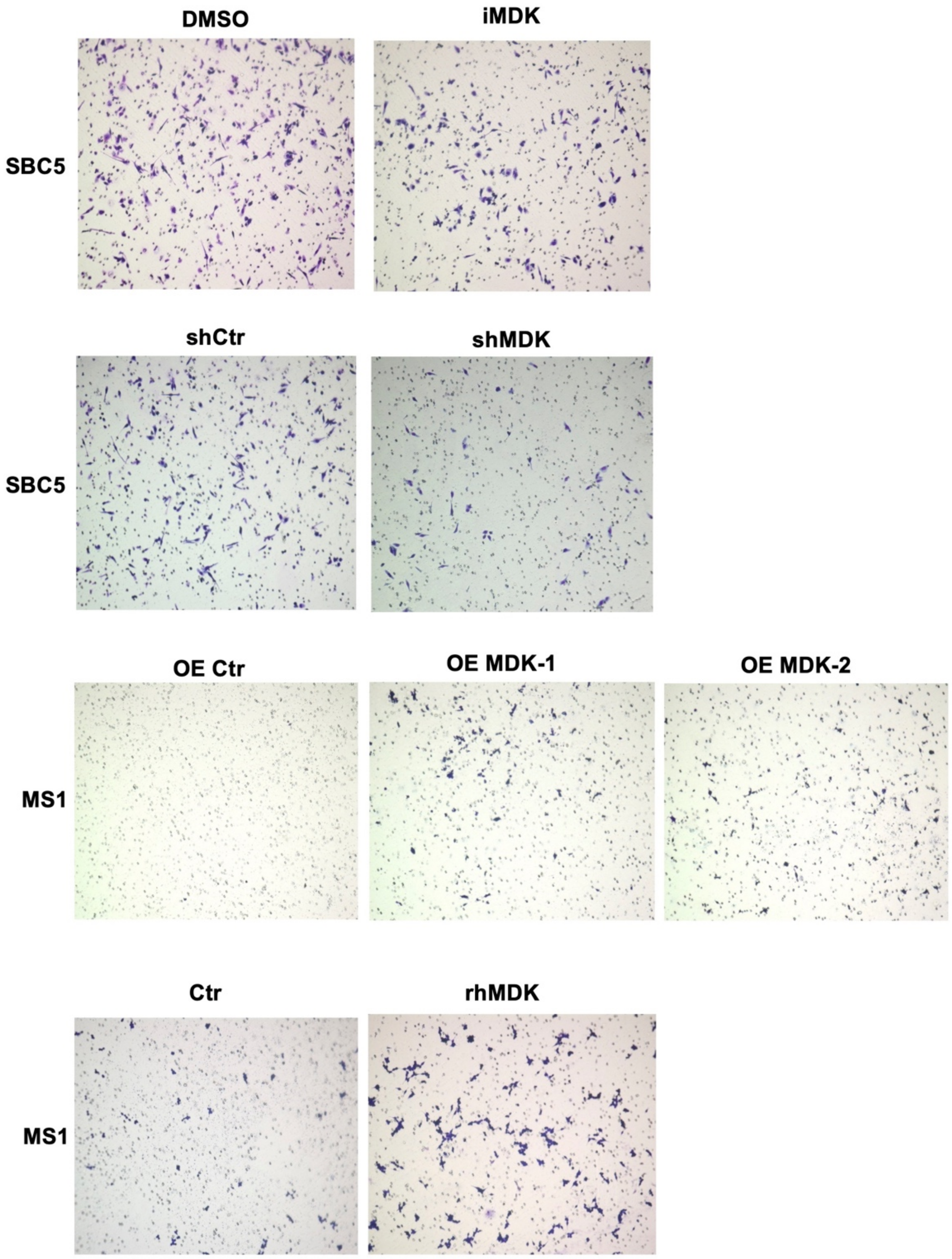

Supplementary Figure 4

Representative images for migration assays

MDK: midkine; Ctr: control; iMDK: MDK inhibitor; shCtr: control shRNA vector; shMDK: shRNA against

MDK; OE: overexpression; rhMDK: recombinant human MDK

Supplementary Figure 5

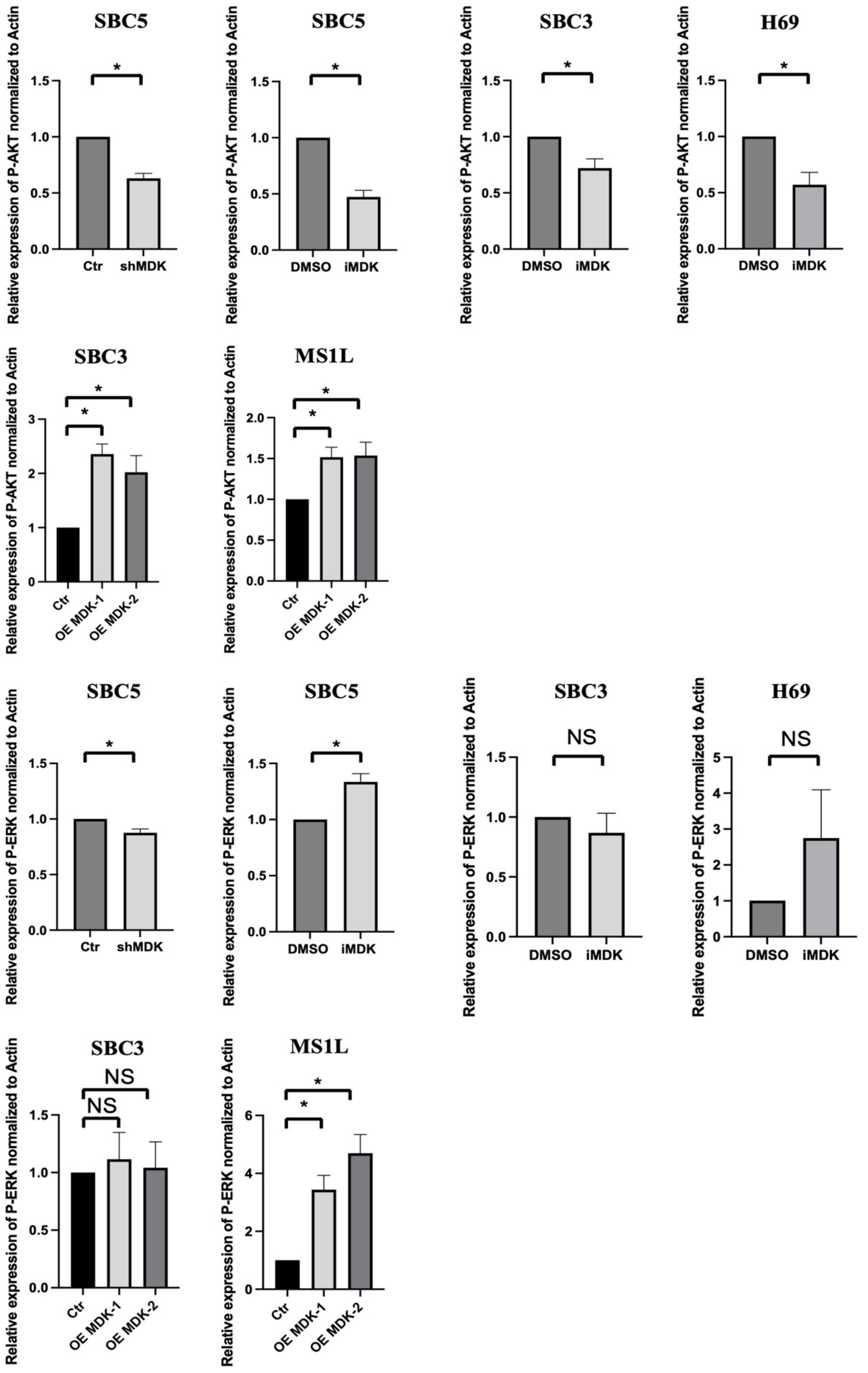

Supplementary Figure 5

As shown in Figure 4A-B, relative changes in P-AKT and P-ERK protein expression in the SCLC cell lines due to changes in MDK expression were quantified and compared. MDK and P-AKT expression was correlated in SCLC cell lines.

*$p < 0.05$; NS: $p > 0.05$. MDK: midkine; Ctr: control; iMDK: MDK inhibitor; shCtr: control shRNA vector; shMDK: shRNA against MDK; OE: overexpression;

Supplementary Figure 6

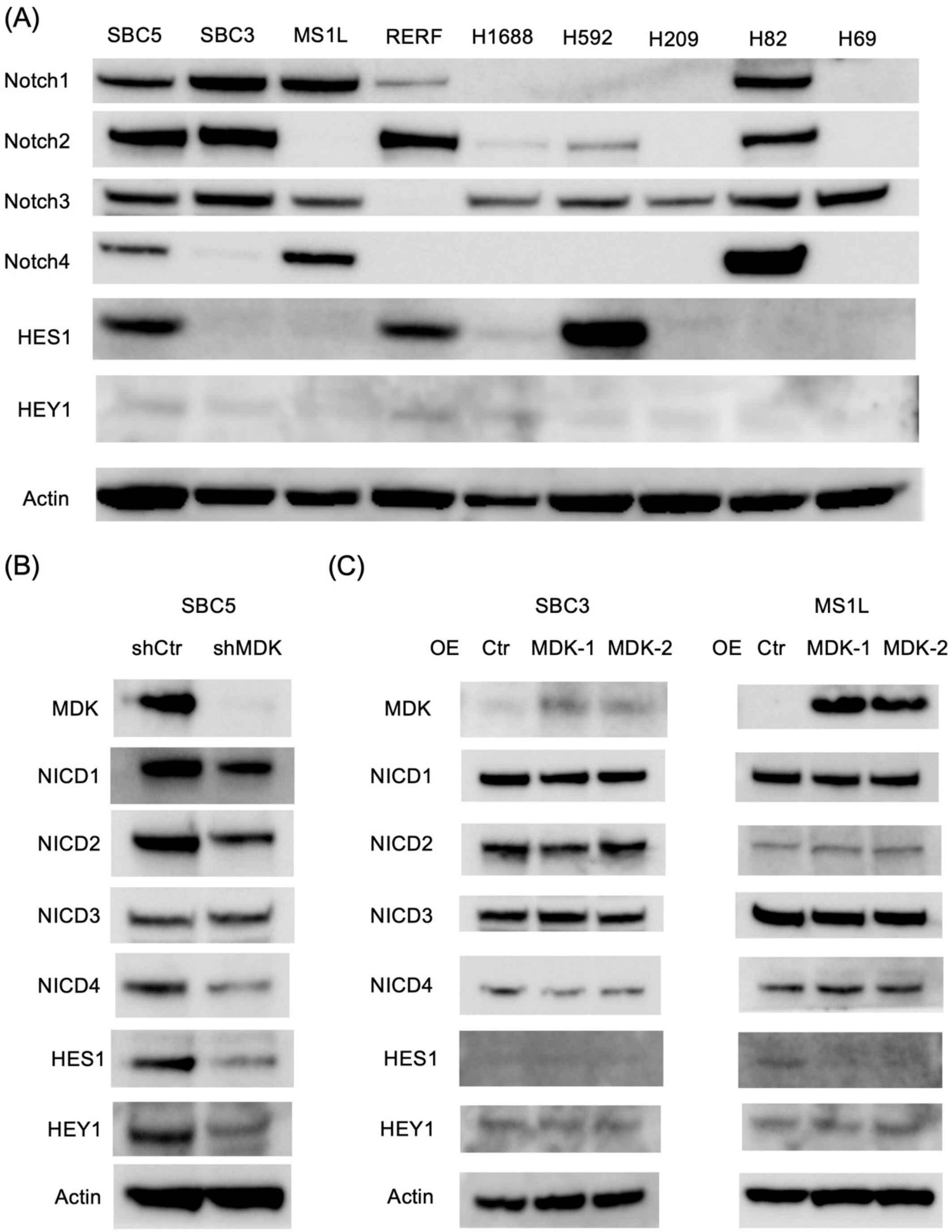

Supplementary Figure 6

MDK may be associated with the Notch pathway in some small cell lung cancer (SCLC) cells

(A) Expression levels of Notch signaling pathway proteins in SCLC cell lines as determined via western blotting.

(B) Expression levels of Notch signaling pathway proteins following MDK knockdown in SBC5 cells as evaluated via western blotting. (C) Expression levels of Notch signaling pathway proteins upon MDK overexpression in SBC3 and MS1L cells as evaluated via western blotting.

MDK: midkine; Notch: neurogenic locus notch homolog protein; NICD: Notch2 intracellular domain expression; HES1: hairy and enhancer of split-1; HEY1: hairy/enhancer-of-split related with YRPW motif protein 1; OE: overexpression; Ctr: control; RERF: RERF-LC-MA cell

Supplementary Figure 7

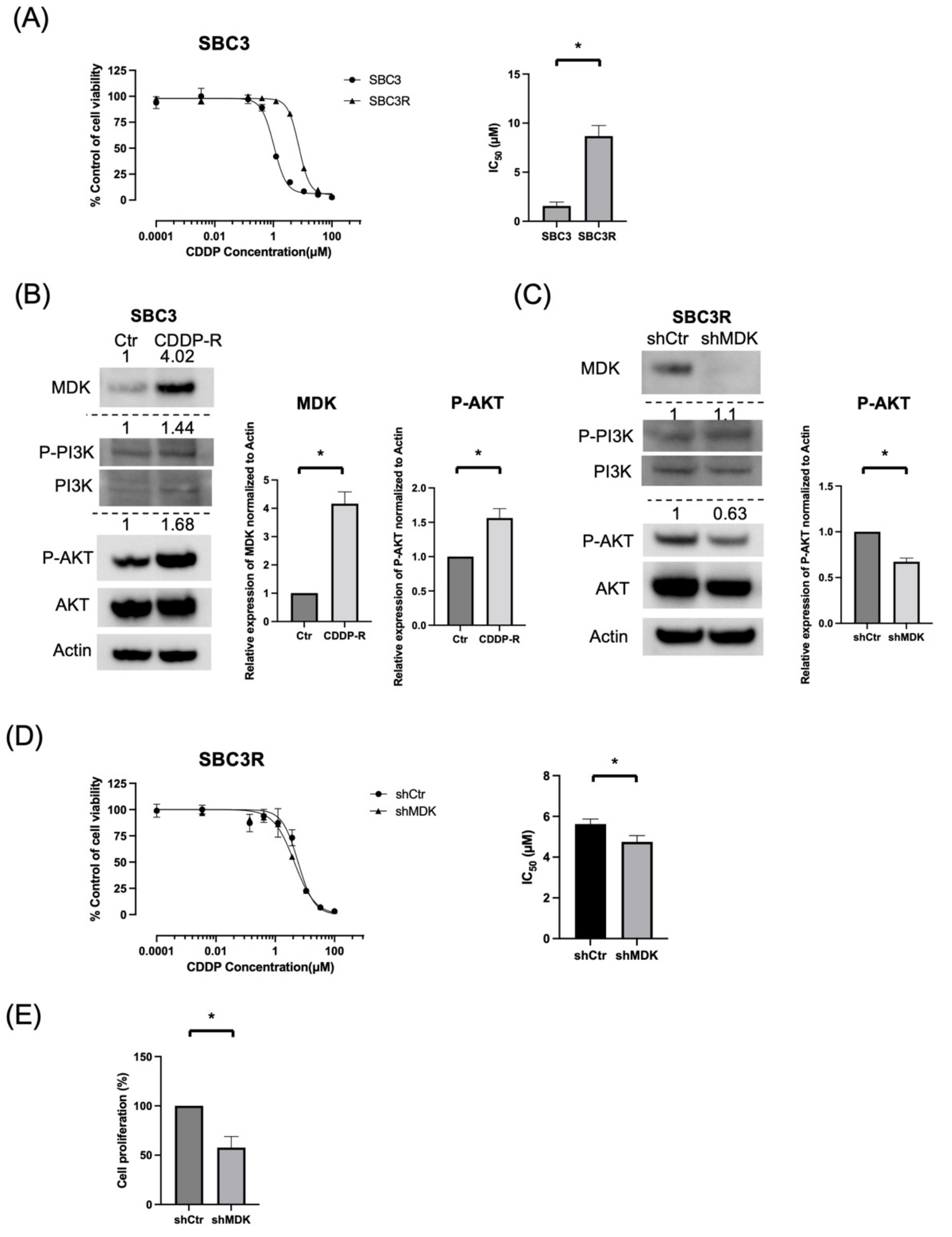

Supplementary Figure 7

MDK and the AKT pathway were activated in small cell lung cancer (SCLC) cells with acquired resistance to CDDP, and MDK knockdown attenuated CDDP resistance via AKT pathway suppression

(A) MTT assay for the CDDP-R SCLC cell lines, SBC3 and SBC3R ($n = 3$; mean ± SD). (B) MDK expression and PI3K/AKT pathway activity in SBC3 and SBC3R cells as determined via western blotting. (C) Changes in MDK expression and PI3K/AKT pathway activity in SBC3R cells following MDK expression suppression using shMDK as determined via western blotting. (D) Changes in the effects of CDDP in SBC3R cells treated with CDDP for 72 h upon MDK knockdown as determined via the MTT assay ($n = 3$; mean ± SD). (E) Cell growth at 96 h in SBC3R cells transfected with shCtr or shMDK after cell seeding as determined via MTT assays ($n = 3$; mean ± SD). *$p < 0.05$; NS, $p > 0.05$. MDK: midkine; Ctr: control; CDDP: cisplatin; CDDP-R: cisplatin resistant; shCtr: control shRNA vector; shMDK: shRNA against MDK; PI3K: phosphatidylinositol 3-kinase; AKT: protein kinase B; P: phosphorylated

Supplementary Figure 8

(A)

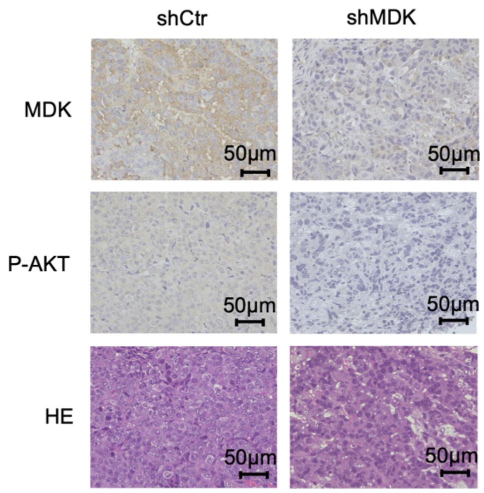

(B)

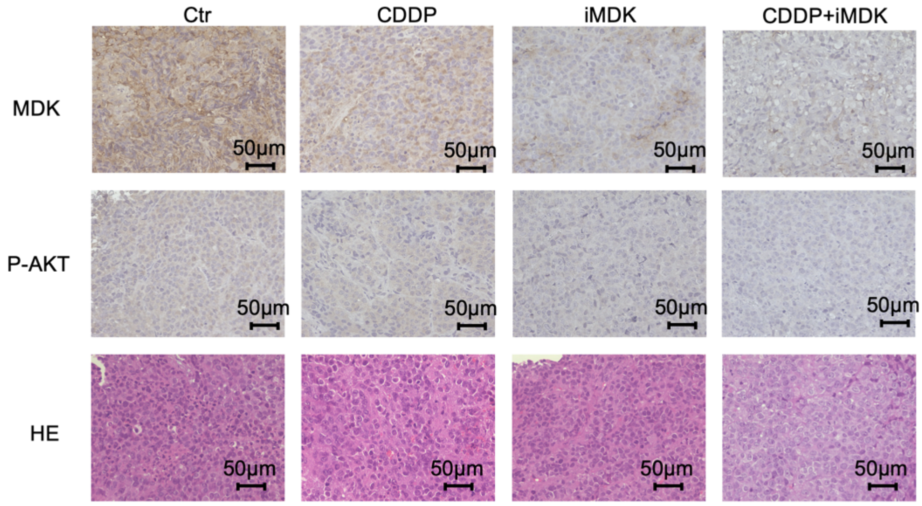

Supplementary Figure 8

Representative images of xenograft tumors subjected to HE and IHC staining for the evaluation of MDK and P-AKT expression

(A) Representative HE and IHC staining images for xenograft tumors generated via subcutaneous injection of SBC5 cells transfected with shCtr or shMDK in nude mice. (B) Representative HE and IHC staining images for xenograft tumors resected two days after drug administration in nude mouse xenograft models generated via subcutaneous SBC5 cell injection. Scale bar: 50 μm. MDK: midkine; Ctr: control; shCtr: control shRNA vector; shMDK: shRNA against MDK; HE: hematoxylin and eosin; IHC: immunohistochemistry; CDDP: cisplatin; iMDK: MDK inhibitor; AKT: protein kinase B; P: phosphorylated
