# Supplementary material for: Midkine Promotes Tumor Growth and Attenuates the Effect of Cisplatin in Small Cell Lung Cancer
Source: Cancer Med. 2025 Jul 7;14(13):e71034. doi: 10.1002/cam4.71034 (PMC12230509; doi:10.1002/cam4.71034)
Supplement: Supplementary file 3 — Tables S1‐S4. [file CAM4-14-e71034-s002.docx]

Table S1

Antibodies used for the immunohistochemical evaluation of clinical samples

| Antibody | Catalog number | Species | Dilution | Manufacturer |
| --- | --- | --- | --- | --- |
| MDK | ab52637 | Rabbit (m) | 1:500 | Abcam |
| ASCL1 | 556604 | Mouse (m) | 1:100 | BD |
| NEUROD1 | ab205300 | Rabbit (m) | 1:50 | Abcam |
| POU2F3 | sc293402 | Mouse (m) | 1:600 | Santa Cruz |
| YAP1 | sc101199 | Mouse (m) | 1:1250 | Santa Cruz |
| SYP | ab32127 | Rabbit (m) | 1:600 | Abcam |
| REST | ab211537 | Mouse (m) | 1:50 | Abcam |
| P-AKT | #4060 | Rabbit (m) | 1:100 | CST |

(m): monoclonal; BD: BD Pharmingen; CST: Cell Signaling Technology

Table S2

Antibodies used for western blot analyses

| Antibody | Catalog number | Species | Dilution | Manufacturer |
| --- | --- | --- | --- | --- |
| MDK | sc-46701 | Mouse (m) | 1:500 | Santa Cruz |
| ASCL1 | 556604 | Mouse (m) | 1:500 | BD |
| NEUROD1 | ab109224 | Rabbit (m) | 1:1000 | abcam |
| POU2F3 | sc-293402 | Mouse (m) | 1:500 | Santa Cruz |
| YAP/TAZ | #8418 | Rabbit (m) | 1:1000 | CST |
| SYP | ab32127 | Rabbit (m) | 1:10000 | abcam |
| REST | LS-C668231 | Rabbit (p) | 1:1000 | LSBio |
| P-PI3K | ab278545 | Rabbit (m) | 1:1000 | abcam |
| Total PI3K | #4257 | Rabbit (m) | 1:1000 | CST |
| P-AKT | #4060 | Rabbit (m) | 1:2000 | CST |
| Total AKT | #4691 | Rabbit (m) | 1:1000 | CST |
| P-ERK1/2 | #4370 | Rabbit (m) | 1:2000 | CST |
| Total ERK1/2 | #4695 | Rabbit (m) | 1:1000 | CST |
| Cleaved PARP | #5625 | Rabbit (m) | 1:1000 | CST |
| Cleaved Caspase3 | #9661 | Rabbit (p) | 1:1000 | CST |
| NICD1 | #3608 | Rabbit (m) | 1:500 | CST |
| NICD2 | #5732 | Rabbit (m) | 1:5000 | CST |
| NICD3 | 55114-1-AP | Rabbit (p) | 1:1000 | Protein tech |
| NICD4 | #2423 | Mouse (m) | 1:1500 | CST |
| HES1 | #11988 | Rabbit (m) | 1:1000 | CST |
| HEY1 | ab154077 | Rabbit (p) | 1:1000 | abcam |
| Actin | A2066 | Rabbit (p) | 1:1500 | Sigma Aldrich |

(m): monoclonal; (p): polyclonal; BD: BD Pharmingen; CST: Cell Signaling Technology; LSBio: Lifespan Biosciences; NICD: Notch intracellular domain

Table S3

IC_50_ values (mean ± SD) for cisplatin and iMDK

| Cell line | IC_50_ of cisplatin (µmol/L) | IC_50_ of iMDK (µmol/L) |
| --- | --- | --- |
| SBC5 | 3.2 ± 0.42 | 0.61 ± 0.16 |
| SBC3 | 1.6 ± 0.31 | 0.60 ± 0.06 |
| H69 | 9.1 ± 0.7 | 2.24 ± 0.15 |
| H82 | 8.6 ± 0.9 | 7.04 ± 0.11 |
| MS1L |  | >1000 |
| SBC5R | 24.8 ± 2.6 |  |
| SBC3R | 8.68 ± 0.89 |  |

Table S4

Changes in IC_50_ values (mean ± SD) for cisplatin following MDK knockdown

| Cell line | sh or OE | IC_50_ of cisplatin (µmol/L) |
| --- | --- | --- |
| SBC5 | sh Ctr | 4.3 ± 0.23 |
|  | sh MDK | 2.1 ± 0.48 |
| SBC3 | OE Ctr | 0.61 ± 0.02 |
|  | OE MDK-1 | 0.69 ± 0.01 |
|  | OE MDK-2 | 0.70 ± 0.03 |
| MS1L | OE Ctr | 8.6 ± 1.7 |
|  | OE MDK-1 | 13.6 ± 1.0 |
|  | OE MDK-2 | 14.3 ± 2.3 |
| SBC5R | sh Ctr | 19.7 ± 0.2 |
|  | sh MDK | 14.5 ± 1.7 |
| SBC3R | sh Ctr | 5.6 ± 0.2 |
|  | sh MDK | 4.8 ± 0.3 |

shCtr: control shRNA vector; shMDK: shRNA against MDK; OE: overexpression
